# Biomedical Signal Processing and Modeling Complexity of Living Systems 2014

**DOI:** 10.1155/2015/567303

**Published:** 2015-03-24

**Authors:** Carlo Cattani, Shengyong Chen, Igor Pantic

**Affiliations:** ^1^Department of Mathematics, University of Salerno, Via Ponte Don Melillo, 84084 Fisciano, Italy; ^2^College of Computer Science & Technology, Zhejiang University of Technology, Hangzhou 310023, China; ^3^Institute of Medical Physiology, University of Belgrade School of Medicine, 11129 Belgrade, Serbia

Living systems are often maintained by information flows, and, as such, they present interesting mathematical problems, for instance, in the modeling and the analysis of spatial structures, self-organization, environmental interaction, behavior, and development. Biomedical signals extract information from the complex phenomena being measured, which are typically a time series having both a regular and random component. Solutions attempt to map general principles, which are used to model how the living systems work. Many researchers have been studying these problems because of their interesting mathematical features and because of their scientific importance. The focus of this special issue is the mathematical analysis and modeling of time series in living systems and biomedical signals. It is mostly interested in the related new development of both theoretical study and practical implementation, with either modeling, complexity, statistics, or signal transformation in living systems.

This special issue includes papers, mainly on these aspects: (1) modeling dynamical complexity in living systems, (2) methods for analysis and characterization of dynamical complexity, (3) biomedical signal analysis, and (4) biomedical image analysis. The selected papers in this special issue represent a good panel in recent challenges. They represent some of the most recent advances in many different investigations devoted to the analysis of complexity in living systems. They cannot be exhaustive because of the rapid growth both of mathematical methods of signal analysis and of the technical performances of devices. However, they aim to offer a wide introduction on a multidisciplinary discipline and to give some of the more interesting and original solution of challenging problems.

There are papers in the category of modeling dynamical complexity in living systems. Y. Ni et al. analyze epileptic seizures using complex network. Y. Yang et al. report a fingerprint encryption scheme based on irreversible function and secure authentication. J. Shen et al. propose a biological hierarchical model for underwater moving object detection. X. Wang et al. give a survey of three-dimensional human motion editing and synthesis.

In the category of methods for analysis of dynamical complexity, nonlinear time series analysis and fractal analysis are often used these years. G. Xu et al. suggest a DAQ-device-based continuous wave near-infrared spectroscopy system for measuring human functional brain activity. H.-T. Wu et al. find the effects of first-time overnight CPAP therapy for increasing the complexity of the patient's physiological system. I. Chifor et al. show mathematical methods for assessing the prognostic of fixed partial dentures resulting from evaluating a group of dental patients in Romania. J. Hu et al. identify odd/even order binary kernel slices for a nonlinear system using inverse repeat m-sequences. M. Zhao et al. propose methods of feature quantification and abnormal detection on cervical squamous epithelial cells. J. Ma et al. propose a method of protein model classification and retrieval using bag-of-visual-features. Q. Guan et al. make three-dimensional simulation of scalp soft tissue expansion using finite element method. J. Zheng et al. show a dimensionality reduction method by supervised neighbor embedding using Laplacian search.

In the category of biomedical signal analysis, G. Vecchiato et al. propose neuroelectrical correlates of trustworthiness and dominance judgments related to the observation of political candidates. S. M. Mousavi et al. use a wavelet transform to determine depth of anesthesia to prevent awareness during general anesthesia. H.-Y. Zheng et al. make a cross-language study on the influence of tone inventory on ERP without focal attention. A. Manosueb et al. report PLI cancellation in ECG signal based on adaptive filter by using Wiener-Hopf equation for providing initial condition.

This special issue also selected some papers on biomedical image analysis. P. L. Rodrigues et al. give automated image analysis of lung branching morphogenesis from microscopic images of fetal rat explants. H. Li et al. show the prediction in computer color matching of dentistry based on GA+BP neural network. R. Fan and X. Jin make controllable edge feature sharpening for dental applications. J. Zhao et al. report craniofacial reconstruction evaluation by geodesic network. Y. Tian et al. study automatic blastomere recognition from a single embryo image. L. Chen et al. propose a hierarchical mergence approach to cell detection in phase contrast microscopy images.

As already mentioned, the topics and papers are not an exhaustive representation of the area of biomedical signal processing and modeling complexity of living systems. However, we believe that we have succeeded to collect some of the most significant papers in this area aiming to improve the scientific debate in this interdisciplinary field.

